# Dynamical behavior of solitons of the (2+1)-dimensional Konopelchenko Dubrovsky system

**DOI:** 10.1038/s41598-023-46593-z

**Published:** 2024-01-02

**Authors:** A. Hussain, T. Parveen, B. A. Younis, Huda U. M. Ahamd, T. F. Ibrahim, Mohammed Sallah

**Affiliations:** 1 Abdus Salam School of Mathematical Sciences, Government College University, Lahore, 54600 Pakistan; 2 Department of Mathematics, Government College University, Faisalabad, 38000 Pakistan; 3 Department of Mathematics, Faculty of Science, King Khalid University, Abha, Saudi Arabia; 4 Department of Mathematics, Faculty of Arts and Science in Sarat Abida, King Khalid University, Abha, Saudi Arabia; 5 Department of Mathematics, Faculty of Sciences and Arts (Mahayel), King Khalid University, Abha, Saudi Arabia; 6 Department of Mathematics, Faculty of Sciences, Mansoura University, Mansoura 35516, Egypt, Mansoura, Egypt; 7 Applied Mathematical Physics Research Group, Physics Department, Faculty of Science, Mansoura University, Mansoura, 35516 Egypt; 8 Department of Physics, College of Sciences, University of Bisha, P.O. Box 344, Bisha, 61922 Saudi Arabia

**Keywords:** Engineering, Mathematics and computing, Optics and photonics, Physics

## Abstract

Utilizing nonlinear evolution equations (NEEs) is common practice to establish the fundamental assumptions underlying natural phenomena. This paper examines the weakly dispersed non-linear waves in mathematical physics represented by the Konopelchenko-Dubrovsky (KD) equations. The $(G^\prime /G^2)$-expansion method is used to analyze the model under consideration. Using symbolic computations, the $(G^\prime /G^2)$-expansion method is used to produce solitary waves and soliton solutions to the $(2+1)$-dimensional KD model in terms of trigonometric, hyperbolic, and rational functions. Mathematica simulations are displayed using two, three, and density plots to demonstrate the obtained solitary wave solutions’ behavior. These proposed solutions have not been documented in the existing literature.

## Introduction

In the field of applied mathematics and physics, NEEs are of enormous importance. Since NEEs address a wide range of phenomena in optical fiber, nonlinear dynamics, fluid mechanics, engineering, plasma physics, and other fields, it is important to find their exact solutions. In addition to its theoretical significance, modeling plays a pivotal role with numerous applications, emerging as a key tool in understanding nonlinear evolution equations. It has evolved into a crucial domain of development, where the exchange and further advancement of various recent mathematical methods contribute to significant progress. The objective of this research is to showcase results and recent developments in the theory of evolution equations, encompassing both theoretical and practical aspects. Many researchers and mathematicians have suggested a variety of efficient methods^1–6^ for finding exact solutions of nonlinear (NPDEs), such as Hirota’s bilinear method^7^, tanh function method^8^, the inverse scattering technique^9^, the Bäcklund transformation method^10^, modified variational iteration method^11^, the multiple exp-function approaches^12^, the Darboux transformation approach^13^, the Lie symmetry analysis^14–16^, the $(G^\prime /G)$-expansion approach^17^, the Kudryashov technique^18^ and the Jacobi elliptic technique^19–21^.

The $(G^\prime /G^2)$-expansion method^22^ is a very efficient, reliable, and simple strategy for locating the numerous soliton-form solutions of various NPDEs. Our understanding and ability to forecast the appropriate structures of the related complex nonlinear systems are made possible by the dynamics of NPDEs. A solitary wave, also known as a soliton, is a particle-like object with finite energy and amplitude that maintains its shape during propagation and restores it after colliding with other solitons. Thus, determining the precise closed-form solutions of NPDEs is a very popular topic in today’s world. The wave-form solutions of such a type of NPDEs are highly advantageous in a variety of fields, including nonlinear sciences, applied mathematics, mathematical physics, engineering, plasma physics, nonlinear dynamics, and applied sciences. The following (2+1)-dimensional KD model will be examined in the present study.

1 $$\begin{gathered} u_{y} = v_{x} \hfill \\ u_{t} - u_{{xxx}} - 6buu_{x} + \frac{3}{2}a^{2} u^{2} u_{x} - 3v_{y} + 3au_{x} v = 0, \hfill \\ \end{gathered}$$

where *a*,  *b* are arbitrary real parameters, *u* and *v* are the dependent variables, and *x*, *y*, *t* are independent variables. This system represents the weakly dispersed non-linear waves in mathematical physics.

Several researchers have investigated the analytical solutions of the KD system using some effective methods. The (2+1)-dimensional KD equation^23^ was developed by Konopelchenko and Dubrovsky in 1984. Wazwaz^24^ documented solutions for this system using hyperbolic, trigonometric, and rational functions. These solutions were transformed into recognizable complex structures such as kinks, solitons, and periodic waves by assigning specific parameter values. Subsequently, Feng et al.^25^ employed the improved Riccati mapping method and the variable separation method to analyze solitary wave solutions, periodic waves, and variable-separation solutions using trigonometric, hyperbolic, and rational functions. Meanwhile, Kumar et al.^26^ utilized the Lie symmetry method to derive periodic waves, singular solutions, cnoidal, and snoidal waves. Ren et al.^27^ utilized the Mobius (conformal invariant form) and truncated Painleve approach to establish nonlocal symmetries. Meanwhile, Alfalqi et al.^28^ employed the B-spline approach and the modified simplest approach involving the KD equation. They outlined solutions, including shock waves, singular solutions, solitary waves, periodic singular waves, and plane waves based on rational, trigonometric, and hyperbolic functions. Khater et al.^29^ used a modified auxiliary equation method to establish analytical traveling wave solutions for this system, encompassing periodic waves, kinks, and solitary waves based on rational, trigonometric, and hyperbolic functions. In a recent approach, Kumar et al.^30^ applied the generalized exponential rational function method and dynamical system method to derive solutions for KD equations, yielding soliton solutions such as kink waves, periodic waves, and oscillating waves based on rational, trigonometric, and hyperbolic functions. In the aforementioned discussion, various approaches were employed to derive solutions for the KD system, yielding trigonometric, hyperbolic, and rational function solutions. Despite sharing the same foundational functions, these solutions exhibited distinct structures, each novel in comparison to the others. Motivated by this, we aim to introduce additional classes of solitary wave solutions for the KD system (1), still rooted in trigonometric, hyperbolic, and rational functions, but with unique structures not yet documented in the literature. This underscores the novelty of our findings.

In this article, the $(G^\prime /G^2)$-expansion method^22,31^ is applied to investigate the (2+1)-dimensional KD model (1). The primary goal is to generate reliable solitary waves and soliton solutions for the KD system (1). The analytical findings are presented in the form of trigonometric, hyperbolic, and rational functions. Additionally, the development of the solitary wave structure is demonstrated through specific soliton-form solutions in two- and three-dimensional graphics, as well as density graphics in Mathematica simulations. The results suggest that the derived exact closed-form solutions exhibit highly impressive and beneficial evolutionary profile dynamics. Notably, the discovered soliton solutions are entirely novel and have not been reported in previous findings.

The structure of this article follows the following plan: The $(G^\prime /G^2)$-expansion method is explained in Section [Sec Sec2]”, which deals with its introduction. Section “[Sec Sec3]” provides the exact traveling wave solution to the KD equations. The dynamics of the wave patterns of the obtained closed-form soliton solutions of the KD equations are covered in Section “Graphical interpretation of some solutions”. In the end, a conclusion is provided.

## Introduction to the $(G^\prime /G^2)$-expansion method

In this section, we give a general overview of ansatz that is utilized to compute the traveling wave structures of some nonlinear equations. Here are the main steps for the ansatz of $(G^\prime /G^2)$-expansion method^22,31^.

**Step 1.** Consider a general system of NPDEs (with two dependent variables and three independent variables) as

2 $$\begin{gathered} K_{1} (u,v,u_{x} ,v_{x} ,u_{t} ,v_{t} ,u_{y} ,v_{y} ,u_{{yy}} ,v_{{yy}} ,u_{{xx}} ,v_{{xx}} ...) = 0 \hfill \\ K_{2} (u,v,u_{x} ,v_{x} ,u_{t} ,v_{t} ,u_{y} ,v_{y} ,u_{{yy}} ,v_{{yy}} ,u_{{xx}} ,v_{{xx}} ...) = 0, \hfill \\ \end{gathered}$$

where *u*, *v* are unknown functions of independent variables $x,\,y,\,t$.

**Step 2.** We apply the following transformation

3 $$\begin{aligned} u(x,y,t)=U(\xi ),\,\,\,v(x,y,t)=U(\xi ) \,\, \text {along with}\,\, \xi =\alpha x+\beta y-\mu t, \end{aligned}$$

to convert (2) into a system of nonlinear ordinary differential equations (NLODES)

4 $$\begin{gathered} H_{1} (U,V,U^{\prime},V, \ldots ) = 0, \hfill \\ H_{2} (U,V,U^{\prime},V, \ldots ) = 0. \hfill \\ \end{gathered}$$

After some mathematical calculations system (4) is converted into a single NLODE

5 $$\begin{aligned} H(U,V,U',V,\ldots )=0, \end{aligned}$$

where $(')$ indicates the differentiation w.r.t. $\xi$.

**Step 3.** We assume that a polynomial in $\left( \frac{G^\prime }{G^2}\right)$ can be used to express the solution of (5) as follows

6 $$\begin{aligned} U(\zeta )=d_{0}+\sum _{n=1}^{N}\bigg (d_{n}\left( \frac{G^{\prime }}{G^2}\right) ^n+d_{-n} \left( \frac{G^{\prime }}{G^2}\right) ^{-n}\bigg ), \end{aligned}$$

where the straightforward Riccati equation is satisfied by $G=G(\xi )$

7 $$\begin{aligned} \left( \frac{G^{\prime }}{G^2}\right) ^{\prime }=\eta +\psi \left( \frac{G^{\prime }}{G^2}\right) ^2. \end{aligned}$$

In the above equation unknown constants $d_{0}$, $d_{n}$, $d_{-n}$ $(n=1,2,\ldots ,M)$ must be found where $\eta \ne 1$ and $\psi \ne 0$ are arbitrary integers.

The three possibilities for the promising solution of $(G^\prime /G^2)$ are listed below;

**Case-i: Trigonometric type solutions**

If we have $\eta \psi >0$, then (7) gives

$$\begin{aligned} \left( \frac{G^{\prime }}{G^2}\right) =\sqrt{\frac{\eta }{\psi }}\bigg (\frac{E_1 \cos \sqrt{\eta \psi }\zeta +E_2 \sin \sqrt{\eta \psi }\zeta }{E_1 \cos \sqrt{\eta \psi }\zeta -E_2 \sin \sqrt{\eta \psi }\zeta }\bigg ) \end{aligned}$$

**Case-ii: Hyperbolic type solutions**

When we have $\eta \psi <0$, then (7) gives

$$\begin{aligned} \left( \frac{G^{\prime }}{G^2}\right) =-\frac{\sqrt{|\eta \psi |}}{\psi }\bigg (\frac{E_1 \sinh (2\sqrt{|\eta \psi |}\zeta )+E_2 \cosh (2\sqrt{|\eta \psi |}\zeta )+E_2}{E_1 \sinh (2\sqrt{|\eta \psi |}\zeta )+E_2 \cosh (2\sqrt{|\eta \psi |}\zeta )-E_2}\bigg ). \end{aligned}$$

**Case-iii: Rational type solutions**

When we have $\eta =0$, $\psi \ne 0$ then from (7) rational function solution can be written as

$$\begin{aligned} \left( \frac{G^{\prime }}{G^2}\right) =\bigg (-\frac{E_1}{\psi (E_1 \zeta +E_2)}\bigg ), \end{aligned}$$

where in all above cases $E_1$ and $E_2$ are constants. In the next section we apply the introduced method.

## Application of the $(G^\prime /G^2)$-expansion method to $(2+1)$-d KD model

The transformations $u(x,y,t)=U(\zeta )$ and $v(x,y,t)=V(\zeta )$, where $\zeta =\alpha x+\beta y-\mu t$ in Eq. (1), lead to the NLODE system presented below.

$$\begin{aligned}{}&\beta U^{\prime }(\zeta )=\alpha V^{\prime }(\zeta ) \\&\quad \frac{3}{2} a^2 \alpha U(\zeta )^2 U^{\prime }(\zeta )+3 a \alpha V(\zeta ) U^{\prime }(\zeta )-6 \alpha b U(\zeta ) U^{\prime }(\zeta )-\mu U^{\prime }(\zeta )-\alpha ^3 U^{(3)}(\zeta )-3 \beta V^{\prime }(\zeta )=0. \end{aligned}$$

The first equation is integrated, and the result is

8 $$\begin{aligned} \beta U(\zeta )=\alpha V(\zeta ). \end{aligned}$$

By incorporating it into the second equation of the NLODEs system and ignoring the integration constant, the following NLODE is produced as

9 $$\begin{aligned} \alpha ^3 U^{\prime \prime }(\zeta )-\frac{a^2}{2} \alpha U^3(\zeta )+\frac{3}{2}(2 \alpha b-a \beta ) U^2(\zeta )+\left( \frac{3 \beta ^2}{\alpha }+\mu \right) U(\zeta )=0. \end{aligned}$$

By applying the balancing principle to the Eq. (9), we get at $M + 2 = 3M$, which results in $M = 1$. Using (6) for $M=1$ formula, we arrive at the trial solution presented as

10 $$\begin{aligned} U(\zeta )=d_{0}+d_{1}\bigg (\frac{G'}{G^2}\bigg )+d_{-1}\bigg (\frac{G'}{G^2}\bigg )^{-1}, \end{aligned}$$

where $d_{0}$, $d_{1}$ and $d_{-1}$ are constants. On inserting (10) and its derivatives along with (7) into (9), we get a system of equations. We obtain the set of algebraic equations by setting the coefficients of powers of $(G^\prime /G^2)$ to zero

11 $$\begin{aligned} &2\alpha ^{3} d_{1} \psi ^{2} - \frac{1}{2}a^{2} \alpha d_{1}^{3} = 0, \\ & - \frac{3}{2}d_{1}^{2} a\beta + 3d_{1}^{2} \alpha b - \frac{3}{2}a^{2} \alpha d_{0} d_{1}^{2} = 0, \\ & \frac{{3\beta ^{2} d_{1} }}{\alpha } + \mu d_{1} + 2\alpha ^{3} d_{1} \psi \eta - \frac{3}{2}a^{2} \alpha d_{0}^{2} d_{1} - \frac{3}{2}a^{2} \alpha d_{1}^{2} d_{{ - 1}} - 3d_{0} d_{1} \alpha b + 6b_{0} b_{1} \alpha b = 0, \\ &- \frac{1}{2}a^{2} \alpha d_{0}^{3} + \frac{{3\beta ^{2} d_{0} }}{\alpha } + 3d_{0}^{2} \alpha b - \frac{3}{2}d_{0}^{2} a\beta - 3a^{2} \alpha d_{0} d_{1} d_{{ - 1}} + \mu d_{0} - 3d_{1} d_{{ - 1}} a\beta + 6d_{1} b_{{ - 1}} \alpha b = 0 \\ & - \frac{1}{2}a^{2} \alpha d_{0}^{3} + \frac{{3\beta ^{2} d_{0} }}{\alpha } + 3d_{0}^{2} \alpha b - \frac{3}{2}d_{0}^{2} a\beta - 3a^{2} \alpha d_{0} d_{1} d_{{ - 1}} + \mu d_{0} - 3d_{1} d_{{ - 1}} a\beta + 6d_{1} b_{{ - 1}} \alpha b = 0, \\& - \frac{3}{2}d_{{ - 1}}^{2} a\beta + 3d_{{ - 1}}^{2} \alpha b - \frac{3}{2}a^{2} \alpha d_{0} d_{{ - 1}}^{2} & = 0, \\ 2\alpha ^{3} d_{{ - 1}} \eta ^{2} - \frac{1}{2}a^{2} \alpha d_{{ - 1}}^{3} = 0. \\ \end{aligned}$$

We obtain the following set of parameters by solving the aforementioned system using the computer program like Mathematica, Maple, or MATLAB.

**Set-1**

12 $$\begin{aligned} \begin{aligned} \alpha&=\alpha ,\,\,\,\beta =\beta ,\,\,\,\eta =\eta ,\,\,\,\mu =-\frac{4\alpha ^4\eta ^2d_{o}^2+3\beta ^2d_{-1}^2}{d_{-1}^2\alpha }, \,\,\,\,\,a=-\frac{2\alpha \eta }{d_{-1}},\,\,\,\,b=\frac{\eta (2\alpha ^2d_{o}\eta -\beta d_{-1})}{d_{-1}^2} \,\,\,\,\,\\ \,\,\,\psi&=\psi ,\,\,\,d_{-1}=d_{-1},\,\,\,\,\,d_{0}=d_{0},\,\,\,\,\,d_{1}=0. \end{aligned} \end{aligned}$$

**Set-2**

13 $$\begin{aligned} \begin{aligned} \alpha&=\alpha ,\,\,\,\beta =\beta ,\,\,\,\eta =\eta ,\,\,\,\mu =-\frac{2\alpha ^4\eta \psi +3\beta ^2}{\alpha }, \,\,\,\,\,a=-\frac{2\alpha \psi }{d_{1}},\,\,\,\,b=\frac{\psi \beta }{d_{1}} \,\,\,\,\,\\ \,\,\,\psi&=\psi ,\,\,\,d_{-1}=0,\,\,\,\,\,d_{0}=0,\,\,\,\,\,d_{1}=d_{1}. \end{aligned} \end{aligned}$$

**Set-3**

14 $$\begin{aligned} \begin{aligned} \alpha&=\alpha ,\,\,\,\beta =\beta ,\,\,\,\eta =-\frac{\psi d_{0}^2}{d_{1}^2},\,\,\,\mu =-\frac{4\alpha ^4\psi ^2d_{0}^2+3\beta ^2d_{1}^2}{d_{1}^2\alpha }, \,\,\,\,\,a=-\frac{2\alpha \psi }{d_{1}},\,\,\,\,b=\frac{\psi (2\alpha ^2d_{0}\psi -\beta d_{1})}{d_{1}^2} \,\,\,\,\,\\ \,\,\,\psi&=\psi ,\,\,\,d_{-1}=0,\,\,\,\,\,d_{0}=d_{0},\,\,\,\,\,d_{1}=d_{1}. \end{aligned} \end{aligned}$$

**Set-4**

15 $$\begin{aligned} \begin{aligned} \alpha&=\alpha ,\,\,\,\beta =\beta ,\,\,\,\eta =-\frac{\psi d_{-1}}{d_{1}},\,\,\,\mu =-\frac{8\alpha ^4\psi ^2d_{-1}-3\beta ^2d_{1}}{\alpha d_{1}}, \,\,\,\,\,a=-\frac{2\alpha \psi }{d_{1}},\,\,\,\,b=\frac{\psi \beta }{d_{1}} \,\,\,\,\,\\ \,\,\,\psi&=\psi ,\,\,\,d_{-1}=d_{-1},\,\,\,\,\,d_{0}=0,\,\,\,\,\,d_{1}=d_{1}. \end{aligned} \end{aligned}$$

**Set-5**

16 $$\begin{aligned} \begin{aligned} \alpha&=\alpha ,\,\,\,\beta =\beta ,\,\,\,\eta =-\frac{1}{2}\frac{a d_{-1}}{\alpha },\,\,\,\mu =\frac{4a\alpha ^3\psi d_{-1}-3\beta ^2}{\alpha }, \,\,\,\,\,a=a,\,\,\,\,b=\frac{1}{2}\frac{a\beta }{\alpha } \,\,\,\,\,\\ \,\,\,\psi&=\psi ,\,\,\,d_{-1}=d_{-1},\,\,\,\,\,d_{0}=0,\,\,\,\,\,d_{1}=\frac{2\alpha \psi }{a}. \end{aligned} \end{aligned}$$

**Set-6**

17 $$\begin{aligned} \begin{aligned} \alpha&=\alpha ,\,\,\,\beta =\beta ,\,\,\,\eta =\frac{2\psi d_{-1}^2}{d_{0}^2},\,\,\,\mu =-\frac{16\alpha ^4\psi ^2 d_{-1}^2+3\beta ^2d_{0}^2}{d_{0}^2\alpha }, \,\,\,\,\,a=\frac{4\alpha \psi d_{-1}}{d_{0}^2},\,\,\,\, \,\,\,\,\,\\ b&=\frac{2\psi d_{-1}(4\alpha ^2\psi d_{-1}+\beta d_{0})}{d_{0}^3}, \,\,\,\psi =\psi ,\,\,\,d_{-1}=d_{-1},\,\,\,\,\,d_{0}=d_{0},\,\,\,\,\,d_{1}=\frac{1}{2}\frac{d_{0}^2}{d_{-1}}\cdot \end{aligned} \end{aligned}$$

**Set-7**

18 $$\begin{aligned} \begin{aligned} \alpha&=\alpha ,\,\,\,\beta =\beta ,\,\,\,\eta =-\frac{4\psi d_{-1}^2}{d_{0}^2},\,\,\,\mu =-\frac{64\alpha ^4\psi ^2 d_{-1}^2+3\beta ^2d_{0}^2}{d_{0}^2\alpha }, \,\,\,\,\,a=\frac{8\alpha \psi d_{-1}}{d_{0}^2},\,\,\,\, \,\,\,\,\,\\ b&=\frac{4\psi d_{-1}(8\alpha ^2\psi d_{-1}+\beta d_{0})}{d_{0}^3}, \,\,\,\psi =\psi ,\,\,\,d_{-1}=d_{-1},\,\,\,\,\,d_{0}=d_{0},\,\,\,\,\,d_{1}=\frac{1}{4}\frac{d_{0}^2}{d_{-1}}\cdot \end{aligned} \end{aligned}$$

The three possibilities for the promising solution of $(G^\prime /G^2)$ from **Set-1** are listed below;

**Case-i: Trigonometric type solutions**

If we have $\eta \psi >0$, then (7) gives

19 $$\begin{aligned} \left( \frac{G^{\prime }}{G^2}\right) =\sqrt{\frac{\eta }{\psi }}\bigg (\frac{E_1 \cos \sqrt{\eta \psi }\zeta +E_2 \sin \sqrt{\eta \psi }\zeta }{E_1 \cos \sqrt{\eta \psi }\zeta -E_2 \sin \sqrt{\eta \psi }\zeta }\bigg ). \end{aligned}$$

**Case-ii: Hyperbolic type solutions**

If we have $\eta \psi <0$, then (7) gives

20 $$\begin{aligned} \left( \frac{G^{\prime }}{G^2}\right) =-\frac{\sqrt{|\eta \psi |}}{\psi }\bigg (\frac{E_1 \sinh (2\sqrt{|\eta \psi |}\zeta )+E_2 \cosh (2\sqrt{|\eta \psi |}\zeta )+E_2}{E_1 \sinh (2\sqrt{|\eta \psi |}\zeta )+E_2 \cosh (2\sqrt{|\eta \psi |}\zeta )-E_2}\bigg ). \end{aligned}$$

**Case-iii: Rational type solutions**

If we have $\eta =0$, $\psi \ne 0$ then from (7) rational solution can be written as

21 $$\begin{aligned} \left( \frac{G^{\prime }}{G^2}\right) =\bigg (-\frac{E_1}{\psi (E_1 \zeta +E_2)}\bigg ), \end{aligned}$$

where $E_1$ and $E_2$ are real parameters.

By back substituting the values in (10), we get the solutions of the system (1) in the form;

**Family-1: Trigonometric type solutions**

If we have $\eta \psi >0$, then

22 $$\begin{aligned} \begin{aligned} u_{1}(x,y,t)&=-\frac{a\beta -2\alpha b}{a^2 \alpha }+d_{-1}\bigg (\sqrt{\frac{\eta }{\psi }}\bigg (\frac{E_1 \cos \sqrt{\eta \psi }\zeta + E_2 \sin \sqrt{\eta \psi }\zeta }{E_1 \cos \sqrt{\eta \psi }\zeta -E_2 \sin \sqrt{\eta \psi }\zeta }\bigg )\bigg )^{-1},\\ v_{1}(x,y,t)&=-\frac{\beta (a\beta -2\alpha b)}{a^2 \alpha ^2}+\frac{\beta }{\alpha }d_{-1}\bigg (\sqrt{\frac{\eta }{\psi }}\bigg (\frac{E_1 \cos \sqrt{\eta \psi }\zeta + E_2 \sin \sqrt{\eta \psi }\zeta }{E_1 \cos \sqrt{\eta \psi }\zeta -E_2 \sin \sqrt{\eta \psi }\zeta }\bigg )\bigg )^{-1}. \end{aligned} \end{aligned}$$

**Family-2: Hyperbolic type solutions**

If we have $\eta \psi <0$, then

23 $$\begin{aligned} \begin{aligned} u_{2}(x,y,t)&=-\frac{a\beta -2\alpha b}{a^2 \alpha }+d_{-1}\bigg (-\frac{\sqrt{|\eta \psi |}}{\psi }\bigg (\frac{E_1 \sinh (2\sqrt{|\eta \psi |}\zeta )+E_2 \cosh (2\sqrt{|\eta \psi |}\zeta )+E_2}{E_1 \sinh (2\sqrt{|\eta \psi |}\zeta )+E_2 \cosh (2\sqrt{|\eta \psi |}\zeta )-E_2}\bigg )\bigg )^{-1},\\ v_{2}(x,y,t)&=-\frac{\beta (a\beta -2\alpha b)}{a^2 \alpha ^2}+\frac{\beta }{\alpha }d_{-1}\bigg (-\frac{\sqrt{|\eta \psi |}}{\psi }\bigg (\frac{E_1 \sinh (2\sqrt{|\eta \psi |}\zeta )+E_2 \cosh (2\sqrt{|\eta \psi |}\zeta )+E_2}{E_1 \sinh (2\sqrt{|\eta \psi |}\zeta )+E_2 \cosh (2\sqrt{|\eta \psi |}\zeta )-E_2}\bigg )\bigg )^{-1}. \end{aligned} \end{aligned}$$

**Family-3: Rational type solutions**

If we have $\eta =0$, $\psi \ne 0$ then rational function solution is given as

24 $$\begin{aligned} \begin{aligned} u_{3}(x,y,t)&= -\frac{a\beta -2\alpha b}{a^2 \alpha }+d_{-1}\bigg (-\frac{E_1}{\psi (E_1 \zeta +E_2)}\bigg )^{-1},\\ v_{3}(x,y,t)&=-\frac{\beta (a\beta -2\alpha b)}{a^2 \alpha ^2}+\frac{\beta }{\alpha }d_{-1}\bigg (-\frac{E_1}{\psi (E_1 \zeta +E_2)}\bigg )^{-1}. \end{aligned} \end{aligned}$$

By substituting the values of ${\textbf {Set-2}}$ in (10), we get the solutions of the system (1) in the form;

**Family-4: Trigonometric type solutions**

If we have $\eta \psi >0$, then

25 $$\begin{aligned} \begin{aligned} u_{4}(x,y,t)&=-\frac{\beta \sigma }{b}\bigg (\sqrt{\frac{\eta }{\psi }}\bigg (\frac{E_1 \cos \sqrt{\eta \psi }\zeta + E_2 \sin \sqrt{\eta \psi }\zeta }{E_1 \cos \sqrt{\eta \psi }\zeta -E_2 \sin \sqrt{\eta \psi }\zeta }\bigg )\bigg )\\&\quad -\frac{\beta \varepsilon }{b}\bigg (\sqrt{\frac{\eta }{\psi }}\bigg (\frac{E_1 \cos \sqrt{\eta \psi }\zeta + E_2 \sin \sqrt{\eta \psi }\zeta }{E_1 \cos \sqrt{\eta \psi }\zeta -E_2 \sin \sqrt{\eta \psi }\zeta }\bigg )\bigg )^{-1},\\ v_{4}(x,y,t)&=-\frac{\beta ^2\sigma }{\alpha b}\bigg (\sqrt{\frac{\eta }{\psi }}\bigg (\frac{E_1 \cos \sqrt{\eta \psi }\zeta + E_2 \sin \sqrt{\eta \psi }\zeta }{E_1 \cos \sqrt{\eta \psi }\zeta -E_2 \sin \sqrt{\eta \psi }\zeta }\bigg )\bigg )\\&\quad -\frac{\beta ^2\varepsilon }{\alpha b}\bigg (\sqrt{\frac{\eta }{\psi }}\bigg (\frac{E_1 \cos \sqrt{\eta \psi }\zeta + E_2 \sin \sqrt{\eta \psi }\zeta }{E_1 \cos \sqrt{\eta \psi }\zeta -E_2 \sin \sqrt{\eta \psi }\zeta }\bigg )\bigg )^{-1}. \end{aligned} \end{aligned}$$

**Family-5: Hyperbolic type solutions**

If we have $\eta \psi <0$, then

26 $$\begin{aligned} \begin{aligned} u_{5}(x,y,t)&=\frac{\beta \sigma }{b}\bigg (\frac{\sqrt{|\eta \psi |}}{\psi }\bigg (\frac{E_1 \sinh (2\sqrt{|\eta \psi |}\zeta )+E_2 \cosh (2\sqrt{|\eta \psi |}\zeta )+E_2}{E_1 \sinh (2\sqrt{|\eta \psi |}\zeta )+E_2 \cosh (2\sqrt{|\eta \psi |}\zeta )-E_2}\bigg )\bigg )\\&\quad -\frac{\beta \varepsilon }{b}\bigg (-\frac{\sqrt{|\eta \psi |}}{\psi }\bigg (\frac{E_1 \sinh (2\sqrt{|\eta \psi |}\zeta )+E_2 \cosh (2\sqrt{|\eta \psi |}\zeta )+E_2}{E_1 \sinh (2\sqrt{|\eta \psi |}\zeta )+E_2 \cosh (2\sqrt{|\eta \psi |}\zeta )-E_2}\bigg )\bigg )^{-1},\\ v_{5}(x,y,t)&=\frac{\beta ^2\sigma }{\alpha b}\bigg (\frac{\sqrt{|\eta \psi |}}{\psi }\bigg (\frac{E_1 \sinh (2\sqrt{|\eta \psi |}\zeta )+E_2 \cosh (2\sqrt{|\eta \psi |}\zeta )+E_2}{E_1 \sinh (2\sqrt{|\eta \psi |}\zeta )+E_2 \cosh (2\sqrt{|\eta \psi |}\zeta )-E_2}\bigg )\bigg )\\&\quad -\frac{\beta ^2\varepsilon }{\alpha b}\bigg (-\frac{\sqrt{|\eta \psi |}}{\psi }\bigg (\frac{E_1 \sinh (2\sqrt{|\eta \psi |}\zeta )+E_2 \cosh (2\sqrt{|\eta \psi |}\zeta )+E_2}{E_1 \sinh (2\sqrt{|\eta \psi |}\zeta )+E_2 \cosh (2\sqrt{|\eta \psi |}\zeta )-E_2}\bigg )\bigg )^{-1}. \end{aligned} \end{aligned}$$

**Family-6: Rational type solutions**

If we have $\eta =0$, $\psi \ne 0$ then rational function solution is given as

27 $$\begin{aligned} \begin{aligned} u_{6}(x,y,t)&=\frac{\beta \sigma }{b}\bigg (\frac{E_1}{\psi (E_1 \zeta +E_2)}\bigg ) -\frac{\beta \varepsilon }{b}\bigg (-\frac{E_1}{\psi (E_1 \zeta +E_2)}\bigg )^{-1},\\ v_{6}(x,y,t)&=\frac{\beta ^2\sigma }{\alpha b}\bigg (\frac{E_1}{\psi (E_1 \zeta +E_2)}\bigg ) -\frac{\beta ^2\varepsilon }{\alpha b}\bigg (-\frac{E_1}{\psi (E_1 \zeta +E_2)}\bigg )^{-1}. \end{aligned} \end{aligned}$$

By substituting the values of ${\textbf {Set-3}}$ in (10), we get the solutions of the system (1) in the form;

**Family-7: Trigonometric type solutions**

If we have $\eta \psi >0$, then

28 $$\begin{aligned} \begin{aligned} u_{7}(x,y,t)&=-\frac{a\beta -2\alpha b}{a^2\alpha }+b_{1}\bigg (\sqrt{\frac{\eta }{\psi }}\bigg (\frac{E_1 \cos \sqrt{\eta \psi }\zeta +E_2 \sin \sqrt{\eta \psi }\zeta }{E_1 \cos \sqrt{\eta \psi }\zeta -E_2 \sin \sqrt{\eta \psi }\zeta }\bigg )\bigg )\\&\quad +\frac{1}{4}\frac{\beta ^2a^2-4a\alpha b\beta +4\alpha ^2b^2}{a^4\alpha ^2b_{1}}\bigg (\sqrt{\frac{\eta }{\psi }}\bigg (\frac{E_1 \cos \sqrt{\eta \psi }\zeta +E_2 \sin \sqrt{\eta \psi }\zeta }{E_1 \cos \sqrt{\eta \psi }\zeta -E_2 \sin \sqrt{\eta \psi }\zeta }\bigg )\bigg )^{-1},\\ v_{7}(x,y,t)&=-\frac{\beta (a\beta -2\alpha b)}{a^2\alpha ^2}+b_{1}\frac{\beta }{\alpha }\bigg (\sqrt{\frac{\eta }{\psi }}\bigg (\frac{E_1 \cos \sqrt{\eta \psi }\zeta +E_2 \sin \sqrt{\eta \psi }\zeta }{E_1 \cos \sqrt{\eta \psi }\zeta -E_2 \sin \sqrt{\eta \psi }\zeta }\bigg )\bigg )\\&\quad +\frac{1}{4}\frac{\beta ^3a^2-4a\alpha b\beta ^2+4\alpha ^2\beta b^2}{a^4\alpha ^3b_{1}}\bigg (\sqrt{\frac{\eta }{\psi }}\bigg (\frac{E_1 \cos \sqrt{\eta \psi }\zeta +E_2 \sin \sqrt{\eta \psi }\zeta }{E_1 \cos \sqrt{\eta \psi }\zeta -E_2 \sin \sqrt{\eta \psi }\zeta }\bigg )\bigg )^{-1}. \end{aligned} \end{aligned}$$

**Family-8: Hyperbolic type solutions**

If we have $\eta \psi <0$, then

29 $$\begin{aligned} \begin{aligned} u_{8}(x,y,t)&=-\frac{a\beta -2\alpha b}{a^2\alpha }-b_{1}\bigg (\frac{\sqrt{|\eta \psi |}}{\psi }\bigg (\frac{E_1 \sinh (2\sqrt{|\eta \psi |}\zeta )+E_2 \cosh (2\sqrt{|\eta \psi |}\zeta )+E_2}{E_1 \sinh (2\sqrt{|\eta \psi |}\zeta )+E_2 \cosh (2\sqrt{|\eta \psi |}\zeta )-E_2}\bigg )\bigg )\\&\quad +\frac{1}{4}\frac{\beta ^2a^2-4a\alpha b\beta +4\alpha ^2b^2}{a^4\alpha ^2b_{1}}\bigg (-\frac{\sqrt{|\eta \psi |}}{\psi }\bigg (\frac{E_1 \sinh (2\sqrt{|\eta \psi |}\zeta )+E_2 \cosh (2\sqrt{|\eta \psi |}\zeta )+E_2}{E_1 \sinh (2\sqrt{|\eta \psi |}\zeta )+E_2 \cosh (2\sqrt{|\eta \psi |}\zeta )-E_2}\bigg )\bigg )^{-1},\\ v_{8}(x,y,t)&=-\frac{\beta (a\beta -2\alpha b)}{a^2\alpha ^2}-b_{1}\frac{\beta }{\alpha }\bigg (\frac{\sqrt{|\eta \psi |}}{\psi }\bigg (\frac{E_1 \sinh (2\sqrt{|\eta \psi |}\zeta )+E_2 \cosh (2\sqrt{|\eta \psi |}\zeta )+E_2}{E_1 \sinh (2\sqrt{|\eta \psi |}\zeta )+E_2 \cosh (2\sqrt{|\eta \psi |}\zeta )-E_2}\bigg )\bigg )\\&\quad +\frac{1}{4}\frac{\beta ^3a^2-4a\alpha b\beta ^2+4\alpha ^2b^2\beta }{a^4\alpha ^3b_{1}}\bigg (-\frac{\sqrt{|\eta \psi |}}{\psi }\bigg (\frac{E_1 \sinh (2\sqrt{|\eta \psi |}\zeta )+E_2 \cosh (2\sqrt{|\eta \psi |}\zeta )+E_2}{E_1 \sinh (2\sqrt{|\eta \psi |}\zeta )+E_2 \cosh (2\sqrt{|\eta \psi |}\zeta )-E_2}\bigg )\bigg )^{-1}. \end{aligned} \end{aligned}$$

**Family-9: Rational type solutions**

If we have $\eta =0$, $\psi \ne 0$ then rational function solution is given as

30 $$\begin{aligned} \begin{aligned} u_{9}(x,y,t)&=-\frac{a\beta -2\alpha b}{a^2\alpha }-b_{1}\bigg (\frac{E_1}{\psi (E_1 \zeta +E_2)}\bigg )+\frac{1}{4}\frac{\beta ^2a^2-4a\alpha b\beta +4\alpha ^2b^2}{a^4\alpha ^2b_{1}}\bigg (-\frac{E_1}{\psi (E_1 \zeta +E_2)}\bigg )^{-1},\\ v_{9}(x,y,t)&=-\frac{\beta (a\beta -2\alpha b)}{a^2\alpha ^2}-b_{1}\frac{\beta }{\alpha }\bigg (\frac{E_1}{\psi (E_1 \zeta +E_2)}\bigg )\\&\quad +\frac{1}{4}\frac{\beta ^3a^2-4a\alpha b\beta ^2+4\alpha ^2\beta b^2}{a^4\alpha ^3b_{1}}\bigg (-\frac{E_1}{\psi (E_1 \zeta +E_2)}\bigg )^{-1}. \end{aligned} \end{aligned}$$

By substituting the values of ${\textbf {Set-4}}$ in (10), we get the solutions of the system (1) in the form;

**Family-10: Trigonometric type solutions**

If we have $\eta \psi >0$, then

31 $$\begin{aligned} \begin{aligned} u_{10}(x,y,t)&=-\frac{a\beta -2\alpha b}{a^2\alpha }+b_{1}\bigg (\sqrt{\frac{\eta }{\psi }}\bigg (\frac{E_1 \cos \sqrt{\eta \psi }\zeta +E_2 \sin \sqrt{\eta \psi }\zeta }{E_1 \cos \sqrt{\eta \psi }\zeta -E_2 \sin \sqrt{\eta \psi }\zeta }\bigg )\bigg ),\\ v_{10}(x,y,t)&=-\frac{\beta (a\beta -2\alpha b)}{a^2\alpha ^2}+b_{1}\frac{\beta }{\alpha }\bigg (\sqrt{\frac{\eta }{\psi }}\bigg (\frac{E_1 \cos \sqrt{\eta \psi }\zeta +E_2 \sin \sqrt{\eta \psi }\zeta }{E_1 \cos \sqrt{\eta \psi }\zeta -E_2 \sin \sqrt{\eta \psi }\zeta }\bigg )\bigg ). \end{aligned} \end{aligned}$$

**Family-11: Hyperbolic type solutions**

When we have $\eta \psi <0$, then

32 $$\begin{aligned} \begin{aligned} u_{11}(x,y,t)&=-\frac{a\beta -2\alpha b}{a^2\alpha }-b_{1}\bigg (\frac{\sqrt{|\eta \psi |}}{\psi }\bigg (\frac{E_1 \sinh (2\sqrt{|\eta \psi |}\zeta )+E_2 \cosh (2\sqrt{|\eta \psi |}\zeta )+E_2}{E_1 \sinh (2\sqrt{|\eta \psi |}\zeta )+E_2 \cosh (2\sqrt{|\eta \psi |}\zeta )-E_2}\bigg )\bigg ),\\ v_{11}(x,y,t)&=-\frac{\beta (a\beta -2\alpha b)}{a^2\alpha ^2}-b_{1}\frac{\beta }{\alpha }\bigg (\frac{\sqrt{|\eta \psi |}}{\psi }\bigg (\frac{E_1 \sinh (2\sqrt{|\eta \psi |}\zeta )+E_2 \cosh (2\sqrt{|\eta \psi |}\zeta )+E_2}{E_1 \sinh (2\sqrt{|\eta \psi |}\zeta )+E_2 \cosh (2\sqrt{|\eta \psi |}\zeta )-E_2}\bigg )\bigg ). \end{aligned} \end{aligned}$$

**Family-12: Rational type solutions**

If we have $\eta =0$, $\psi \ne 0$ then rational function solution is given as

33 $$\begin{aligned} \begin{aligned} u_{12}(x,y,t)&=-\frac{a\beta -2\alpha b}{a^2\alpha }-b_{1}\bigg (\frac{E_1}{\psi (E_1 \zeta +E_2)}\bigg ),\\ v_{12}(x,y,t)&=-\frac{\beta (a\beta -2\alpha b)}{a^2\alpha ^2}-b_{1}\frac{\beta }{\alpha }\bigg (\frac{E_1}{\psi (E_1 \zeta +E_2)}\bigg ). \end{aligned} \end{aligned}$$

By substituting the values of ${\textbf {Set-5}}$ in (10), we get the solutions of the system (1) in the form;

**Family-13: Trigonometric type solutions**

When we have $\eta \psi >0$, then

34 $$\begin{aligned} \begin{aligned} u_{13}(x,y,t)&=\frac{2\alpha \psi }{a}\bigg (\sqrt{\frac{\eta }{\psi }}\bigg (\frac{E_1 \cos \sqrt{\eta \psi }\zeta + E_2 \sin \sqrt{\eta \psi }\zeta }{E_1 \cos \sqrt{\eta \psi }\zeta -E_2 \sin \sqrt{\eta \psi }\zeta }\bigg )\bigg )\\&\quad +d_{-1}\bigg (\sqrt{\frac{\eta }{\psi }}\bigg (\frac{E_1 \cos \sqrt{\eta \psi }\zeta + E_2 \sin \sqrt{\eta \psi }\zeta }{E_1 \cos \sqrt{\eta \psi }\zeta -E_2 \sin \sqrt{\eta \psi }\zeta }\bigg )\bigg ),\\ v_{13}(x,y,t)&=\frac{2\beta \psi }{a}\bigg (\sqrt{\frac{\eta }{\psi }}\bigg (\frac{E_1 \cos \sqrt{\eta \psi }\zeta + E_2 \sin \sqrt{\eta \psi }\zeta }{E_1 \cos \sqrt{\eta \psi }\zeta -E_2 \sin \sqrt{\eta \psi }\zeta }\bigg )\bigg )\\&\quad +d_{-1}\frac{\beta }{\alpha }\bigg (\sqrt{\frac{\eta }{\psi }}\bigg (\frac{E_1 \cos \sqrt{\eta \psi }\zeta + E_2 \sin \sqrt{\eta \psi }\zeta }{E_1 \cos \sqrt{\eta \psi }\zeta -E_2 \sin \sqrt{\eta \psi }\zeta }\bigg )\bigg ). \end{aligned} \end{aligned}$$

**Family-14: Hyperbolic type solutions**

When we have $\eta \psi <0$, then

35 $$\begin{aligned} \begin{aligned} u_{14}(x,y,t)&=\frac{2\alpha \psi }{a}\bigg (-\frac{\sqrt{|\eta \psi |}}{\psi }\bigg (\frac{E_1 \sinh (2\sqrt{|\eta \psi |}\zeta )+E_2 \cosh (2\sqrt{|\eta \psi |}\zeta )+E_2}{E_1 \sinh (2\sqrt{|\eta \psi |}\zeta )+E_2 \cosh (2\sqrt{|\eta \psi |}\zeta )-F}\bigg )\bigg )\\&\quad +d_{-1}\bigg (-\frac{\sqrt{|\eta \psi |}}{\psi }\bigg (\frac{E_1 \sinh (2\sqrt{|\eta \psi |}\zeta )+E_2 \cosh (2\sqrt{|\eta \psi |}\zeta )+E_2}{E_1 \sinh (2\sqrt{|\eta \psi |}\zeta )+E_2 \cosh (2\sqrt{|\eta \psi |}\zeta )-E_2}\bigg )\bigg )^{-1},\\ v_{14}(x,y,t)&=\frac{2\beta \psi }{a}\bigg (-\frac{\sqrt{|\eta \psi |}}{\psi }\bigg (\frac{E_1 \sinh (2\sqrt{|\eta \psi |}\zeta )+E_2 \cosh (2\sqrt{|\eta \psi |}\zeta )+E_2}{E_1 \sinh (2\sqrt{|\eta \psi |}\zeta )+E_2 \cosh (2\sqrt{|\eta \psi |}\zeta )-F}\bigg )\bigg )\\&\quad +d_{-1}\frac{\beta }{\alpha }\bigg (-\frac{\sqrt{|\eta \psi |}}{\psi }\bigg (\frac{E_1 \sinh (2\sqrt{|\eta \psi |}\zeta )+E_2 \cosh (2\sqrt{|\eta \psi |}\zeta )+E_2}{E_1 \sinh (2\sqrt{|\eta \psi |}\zeta )+E_2 \cosh (2\sqrt{|\eta \psi |}\zeta )-F}\bigg )\bigg )^{-1}. \end{aligned} \end{aligned}$$

**Family-15: Rational type solutions**

When we have $\eta =0$, $\psi \ne 0$ then rational function solution is given as

36 $$\begin{aligned} \begin{aligned} u_{15}(x,y,t)&=\frac{2\alpha \psi }{a}\bigg (-\frac{E_1}{\psi (E_1 \zeta +E_2)}\bigg )+d_{-1}\bigg (-\frac{E_1}{\psi (E_1 \zeta +E_2)}\bigg )^{-1},\\ v_{15}(x,y,t)&=\frac{2\beta \psi }{a}\bigg (-\frac{E_1}{\psi (E_1 \zeta +E_2)}\bigg )+d_{-1}\frac{\beta }{\alpha }\bigg (-\frac{E_1}{\psi (E_1 \zeta +E_2)}\bigg )^{-1}. \end{aligned} \end{aligned}$$

By substituting the values of ${\textbf {Set-6}}$ in (10), we get the solutions of the system (1) in the form;

**Family-16: Trigonometric type solutions**

If we have $\eta \psi >0$, then

37 $$\begin{aligned} \begin{aligned} u_{16}(x,y,t)&=d_{0}+\frac{1}{2}\frac{d_{0}^2}{d_{-1}}\bigg (\sqrt{\frac{\eta }{\psi }}\bigg (\frac{E_1 \cos \sqrt{\eta \psi }\zeta + E_2 \sin \sqrt{\eta \psi }\zeta }{E_1 \cos \sqrt{\eta \psi }\zeta -E_2 \sin \sqrt{\eta \psi }\zeta }\bigg )\bigg )\\&\quad +d_{-1}\bigg (\sqrt{\frac{\eta }{\psi }}\bigg (\frac{E_1 \cos \sqrt{\eta \psi }\zeta + E_2 \sin \sqrt{\eta \psi }\zeta }{E_1 \cos \sqrt{\eta \psi }\zeta -E_2 \sin \sqrt{\eta \psi }\zeta }\bigg )\bigg )^{-1},\\ v_{16}(x,y,t)&=d_{0}\frac{\beta }{\alpha }+\frac{1}{2}\frac{d_{0}^2\beta }{\alpha d_{-1}}\bigg (\sqrt{\frac{\eta }{\psi }}\bigg (\frac{E_1 \cos \sqrt{\eta \psi }\zeta + E_2 \sin \sqrt{\eta \psi }\zeta }{E_1 \cos \sqrt{\eta \psi }\zeta -E_2 \sin \sqrt{\eta \psi }\zeta }\bigg )\bigg )\\&\quad +d_{-1}\frac{\beta }{\alpha }\bigg (\sqrt{\frac{\eta }{\psi }}\bigg (\frac{E_1 \cos \sqrt{\eta \psi }\zeta + E_2 \sin \sqrt{\eta \psi }\zeta }{E_1 \cos \sqrt{\eta \psi }\zeta -E_2 \sin \sqrt{\eta \psi }\zeta }\bigg )\bigg )^{-1}. \end{aligned} \end{aligned}$$

**Family-17: Hyperbolic type solutions**

When we have $\eta \psi <0$, then

38 $$\begin{aligned} \begin{aligned} u_{17}(x,y,t)&=d_{0}+\frac{1}{2}\frac{d_{0}^2}{d_{-1}}\bigg (-\frac{\sqrt{|\eta \psi |}}{\psi }\bigg (\frac{E_1 \sinh (2\sqrt{|\eta \psi |}\zeta )+E_2 \cosh (2\sqrt{|\eta \psi |}\zeta )+E_2}{E_1 \sinh (2\sqrt{|\eta \psi |}\zeta )+E_2 \cosh (2\sqrt{|\eta \psi |}\zeta )-E_2}\bigg )\\&\quad +d_{-1}\bigg (-\frac{\sqrt{|\eta \psi |}}{\psi }\bigg (\frac{E_1 \sinh (2\sqrt{|\eta \psi |}\zeta )+E_2 \cosh (2\sqrt{|\eta \psi |}\zeta )+E_2}{E_1 \sinh (2\sqrt{|\eta \psi |}\zeta )+E_2 \cosh (2\sqrt{|\eta \psi |}\zeta )-E_2}\bigg )^{-1},\\ v_{17}(x,y,t)&=d_{0}\frac{\beta }{\alpha }+\frac{1}{2}\frac{\beta d_{0}^2}{\alpha d_{-1}}\bigg (-\frac{\sqrt{|\eta \psi |}}{\psi }\bigg (\frac{E_1 \sinh (2\sqrt{|\eta \psi |}\zeta )+E_2 \cosh (2\sqrt{|\eta \psi |}\zeta )+E_2}{E_1 \sinh (2\sqrt{|\eta \psi |}\zeta )+E_2 \cosh (2\sqrt{|\eta \psi |}\zeta )-F}\bigg )\\&\quad +d_{-1}\frac{\beta }{\alpha }\bigg (-\frac{\sqrt{|\eta \psi |}}{\psi }\bigg (\frac{E_1 \sinh (2\sqrt{|\eta \psi |}\zeta )+E_2 \cosh (2\sqrt{|\eta \psi |}\zeta )+E_2}{E_1 \sinh (2\sqrt{|\eta \psi |}\zeta )+E_2 \cosh (2\sqrt{|\eta \psi |}\zeta )-E_2}\bigg )^{-1}. \end{aligned} \end{aligned}$$

**Family-18: Rational type solutions**

If we have $\eta =0$, $\psi \ne 0$ then rational function solution is given as

39 $$\begin{aligned} \begin{aligned} u_{18}(x,y,t)&=d_{0}+\frac{1}{2}\frac{d_{0}^2}{d_{-1}}\bigg (-\frac{E_1}{\psi (E_1 \zeta +E_2)}\bigg )+d_{-1}\bigg (-\frac{E_1}{\psi (E_1 \zeta +E_2)}\bigg )^{-1},\\ v_{18}(x,y,t)&=d_{0}\frac{\beta }{\alpha }+\frac{1}{2}\frac{\beta d_{0}^2}{\alpha d_{-1}}\bigg (-\frac{E_1}{\psi (E_1 \zeta +E_2)}\bigg )+d_{-1}\frac{\beta }{\alpha }\bigg (-\frac{E_1}{\psi (E_1 \zeta +E_2)}\bigg )^{-1}. \end{aligned} \end{aligned}$$

By substituting the values of ${\textbf {Set-7}}$ in (10), we get the solutions of the system (1) in the form;

**Family-19: Trigonometric type solutions**

If we have $\eta \psi >0$, then

40 $$\begin{aligned} \begin{aligned} u_{19}(x,y,t)&=d_{0}+\frac{1}{4}\frac{d_{0}^2}{d_{-1}}\bigg (\sqrt{\frac{\eta }{\psi }}\bigg (\frac{E_1 \cos \sqrt{\eta \psi }\zeta +E_2 \sin \sqrt{\eta \psi }\zeta }{E_1 \cos \sqrt{\eta \psi }\zeta -E_2 \sin \sqrt{\eta \psi }\zeta }\bigg )\bigg )\\&\quad +d_{-1}\bigg (\sqrt{\frac{\eta }{\psi }}\bigg (\frac{E_1 \cos \sqrt{\eta \psi }\zeta +E_2 \sin \sqrt{\eta \psi }\zeta }{E_1 \cos \sqrt{\eta \psi }\zeta -E_2 \sin \sqrt{\eta \psi }\zeta }\bigg )\bigg )^{-1},\\ v_{19}(x,y,t)&=d_{0}\frac{\beta }{\alpha }+\frac{1}{4}\frac{\beta d_{0}^2}{\alpha d_{-1}}\bigg (\sqrt{\frac{\eta }{\psi }}\bigg (\frac{E_1 \cos \sqrt{\eta \psi }\zeta +E_2 \sin \sqrt{\eta \psi }\zeta }{E_1 \cos \sqrt{\eta \psi }\zeta -E_2 \sin \sqrt{\eta \psi }\zeta }\bigg )\bigg )\\&\quad +d_{-1}\frac{\beta }{\alpha }\bigg (\sqrt{\frac{\eta }{\psi }}\bigg (\frac{E_1 \cos \sqrt{\eta \psi }\zeta +E_2 \sin \sqrt{\eta \psi }\zeta }{E_1 \cos \sqrt{\eta \psi }\zeta -E_2 \sin \sqrt{\eta \psi }\zeta }\bigg )\bigg )^{-1}. \end{aligned} \end{aligned}$$

**Family-20: Hyperbolic type solutions**

When we have $\eta \psi <0$, then

41 $$\begin{aligned} \begin{aligned} u_{20}(x,y,t)&=d_{0}+\frac{1}{4}\frac{d_{0}^2}{d_{-1}}\bigg (-\frac{\sqrt{|\eta \psi |}}{\psi }\bigg (\frac{E_1 \sinh (2\sqrt{|\eta \psi |}\zeta )+E_2 \cosh (2\sqrt{|\eta \psi |}\zeta )+E_2}{E_1 \sinh (2\sqrt{|\eta \psi |}\zeta )+E_2 \cosh (2\sqrt{|\eta \psi |}\zeta )-F}\bigg )\bigg )\\&\quad +d_{-1}\bigg (-\frac{\sqrt{|\eta \psi |}}{\psi }\bigg (\frac{E_1 \sinh (2\sqrt{|\eta \psi |}\zeta )+E_2 \cosh (2\sqrt{|\eta \psi |}\zeta )+E_2}{E_1 \sinh (2\sqrt{|\eta \psi |}\zeta )+E_2 \cosh (2\sqrt{|\eta \psi |}\zeta )-E_2}\bigg )\bigg )^{-1},\\ v_{20}(x,y,t)&=d_{0}\frac{\beta }{\alpha }+\frac{1}{4}\frac{\beta d_{0}^2}{\alpha d_{-1}}\bigg (-\frac{\sqrt{|\eta \psi |}}{\psi }\bigg (\frac{E_1 \sinh (2\sqrt{|\eta \psi |}\zeta )+E_2 \cosh (2\sqrt{|\eta \psi |}\zeta )+E_2}{E_1 \sinh (2\sqrt{|\eta \psi |}\zeta )+E_2 \cosh (2\sqrt{|\eta \psi |}\zeta )-F}\bigg )\bigg )\\&\quad +d_{-1}\frac{\beta }{\alpha }\bigg (-\frac{\sqrt{|\eta \psi |}}{\psi }\bigg (\frac{E_1 \sinh (2\sqrt{|\eta \psi |}\zeta )+E_2 \cosh (2\sqrt{|\eta \psi |}\zeta )+E_2}{E_1 \sinh (2\sqrt{|\eta \psi |}\zeta )+E_2 \cosh (2\sqrt{|\eta \psi |}\zeta )-E_2}\bigg )\bigg )^{-1}. \end{aligned} \end{aligned}$$

**Family-21: Rational type solutions**

If we have $\eta =0$, $\psi \ne 0$ then rational function solution is given as

42 $$\begin{aligned} \begin{aligned} u_{21}(x,y,t)&=d_{0}+\frac{1}{4}\frac{d_{0}^2}{d_{-1}}\bigg (-\frac{E_1}{\psi (E_1 \zeta +E_2)}\bigg )+ d_{-1}\bigg (-\frac{E_1}{\psi (E_1 \zeta +E_2)}\bigg )^{-1},\\ v_{21}(x,y,t)&=d_{0}\frac{\beta }{\alpha }+\frac{1}{4}\frac{\beta d_{0}^2}{\alpha d_{-1}}\bigg (-\frac{E_1}{\psi (E_1 \zeta +E_2)}\bigg )+ d_{-1}\frac{\beta }{\alpha }\bigg (-\frac{E_1}{\psi (E_1 \zeta +E_2)}\bigg )^{-1}, \end{aligned} \end{aligned}$$

where in all above families $\zeta =\alpha x+\beta y-\mu t$ and $E_1,\,\, E_2$ are constants.

## Graphical interpretation of some solutions

**Figure 1:**
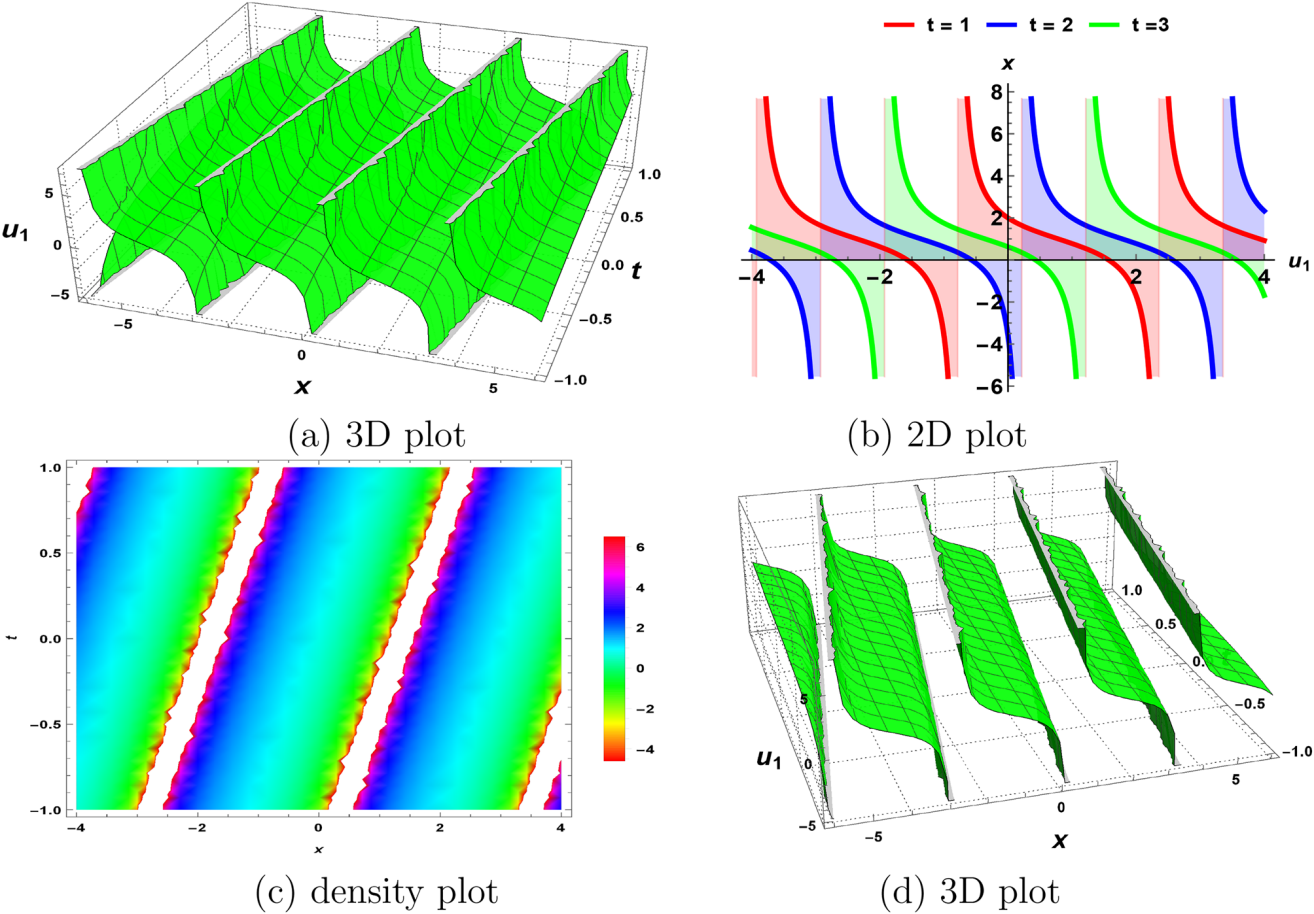
The nature of solitary wave solution (22) obtained by fixing all parameters to 1.

**Figure 2:**
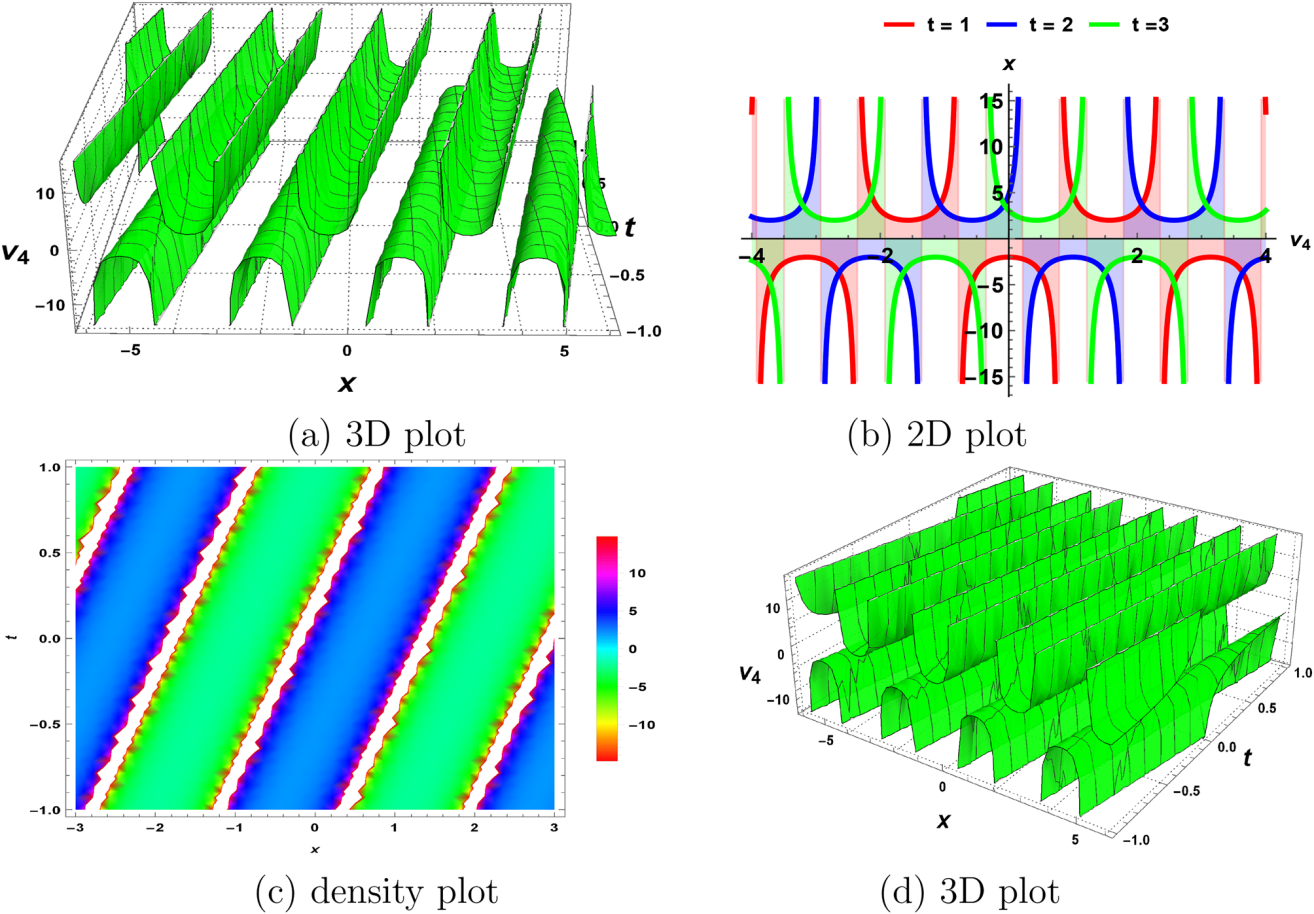
The nature of periodic waves (25) obtained by fixing all parameters to 1.

**Figure 3:**
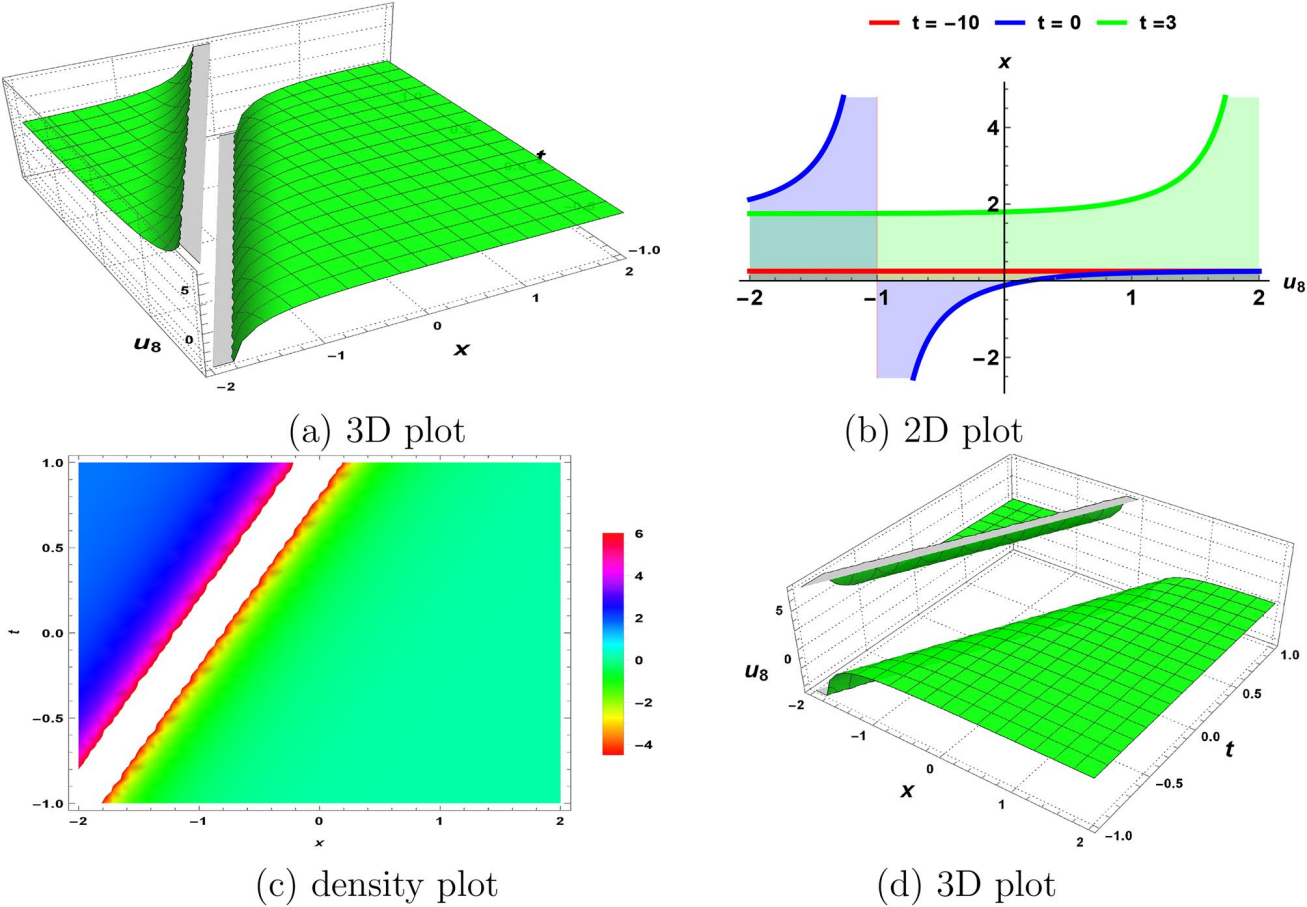
The nature of solitary wave solution (29) obtained by fixing all parameters to 1.

**Figure 4:**
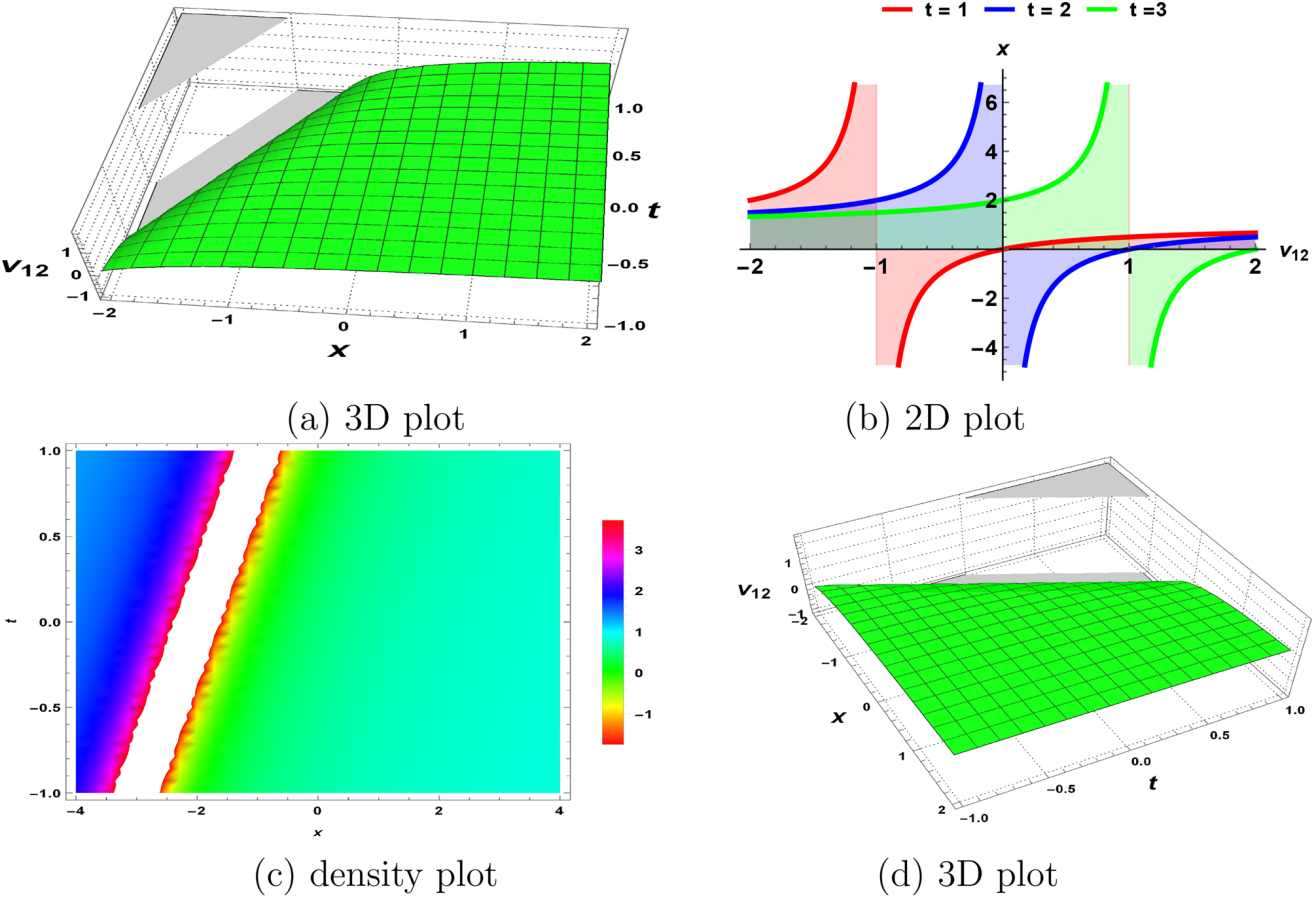
The wave nature of rational polynomial solution (33) obtained by fixing all parameters to 1.

We can demonstrate the graphical representation of the wave solution profile of various solution surfaces in this section. Using symbolic computations, the $(G^\prime /G^2)$-expansion is used to produce waveform soliton solutions to the $(2+1)$-dimensional KD model in terms of trigonometric, hyperbolic, and rational function solutions. Figures 1, 2, 3 and 4 show the periodic waves as well as solitary wave solutions for the KD system (1). An essential set of parameters that are mentioned with each case are included in each plot.

## Discussion and conclusions

It is common practice to utilize NEEs to establish the fundamental assumptions underlying natural phenomena. In this paper, the weakly dispersed non-linear waves in mathematical physics were represented by the KD equations. The $(G^\prime /G^2)$-expansion method was used to analyze the model under consideration. Several researchers have obtained the analytical solutions of the KD system using hyperbolic, trigonometric, and rational functions. In summarizing previous work, Wazwaz^24^ reported the solutions, introducing kink, soliton, and periodic wave solutions. Subsequently, Feng et al.^25^ explored solitary wave, periodic wave, and variable-separation solutions. Concurrently, Kumar et al.^26^ derived periodic waves, singular solutions, cnoidal, and snoidal waves. Additionally, Alfalqi et al.^28^ outlined solutions, including shock waves, singular solutions, solitary waves, periodic singular waves, and plane waves. Khater et al.^29^ established periodic waves, kinks, and solitary waves. In an approach, Kumar et al.^30^ obtained soliton solutions such as kink waves, periodic waves, and oscillating waves. Although various approaches in the discussion produced trigonometric, hyperbolic, and rational function solutions with distinct structures, our approach also explored the same class of rational, hyperbolic, and trigonometric function-based solutions. Comparing results, we conclude that the literature has not featured any of our produced solutions. The obtained solitary wave families validate the method, showcasing applications in solitary wave theory, mathematical sciences, and nonlinear sciences. For future studies, we intend to investigate the KD system using the Jacobi elliptic function method to derive elliptic function-based solutions.

## Data Availability

All data generated or analyzed during this study are included in this published article [and its supplementary information files].
